# Bayesian criterion‐based assessments of recurrent event models with applications to commercial truck driver behavior studies

**DOI:** 10.1002/sim.9528

**Published:** 2022-07-24

**Authors:** Yiming Zhang, Ming‐Hui Chen, Feng Guo

**Affiliations:** ^1^ Department of Statistics University of Connecticut Storrs Connecticut; ^2^ Department of Statistics Virginia Tech Blacksburg Virginia

**Keywords:** Bayesian model assessment, concordance index, multitype recurrent event, penalized spline, truck driving safety

## Abstract

Multitype recurrent events are commonly observed in transportation studies, since commercial truck drivers may encounter different types of safety critical events (SCEs) and take different lengths of on‐duty breaks in a driving shift. Bayesian nonhomogeneous Poisson process models are a flexible approach to jointly model the intensity functions of the multitype recurrent events. For evaluating and comparing these models, the deviance information criterion (DIC) and the logarithm of the pseudo‐marginal likelihood (LPML) are studied and Monte Carlo methods are developed for computing these model assessment measures. We also propose a set of new concordance indices (C‐indices) to evaluate various discrimination abilities of a Bayesian multitype recurrent event model. Specifically, the within‐event C‐index quantifies adequacy of a given model in fitting the recurrent event data for each type, the between‐event C‐index provides an assessment of the model fit between two types of recurrent events, and the overall C‐index measures the model's discrimination ability among multiple types of recurrent events simultaneously. Moreover, we jointly model the incidence of SCEs and on‐duty breaks with driving behaviors using a Bayesian Poisson process model with time‐varying coefficients and time‐dependent covariates. An in‐depth analysis of a real dataset from the commercial truck driver naturalistic driving study is carried out to demonstrate the usefulness and applicability of the proposed methodology.

## 1 INTRODUCTION

Commercial truck crashes are a major traffic safety concern, as there are more than 4000 fatalities and more than 100 000 injuries annually in the United States. Among many crash risk factors, driver‐related factors are the largest contributor, bearing responsibility for the majority of crashes.^1^ Commercial truck drivers are professionals with revenue‐generating driving tasks that require extended working and driving hours. The fatigue and health issues associated with long service hours are a major threat to safety and well‐being of truck drivers. Understanding drivers' sleeping patterns, driving and working schedules, and rest breaks can provide valuable insight into their behaviors, help in developing safety countermeasures, and support safety regulations.

As professional drivers, commercial truck drivers are subject to the operation and safety management of the fleet they serve. Their schedules are influenced by the demand of clients and fleet operation characteristics. In addition, Hours‐of‐Service (HOS) rules set by the Federal Motor Carrier Safety Administration limit the total working hours, total driving time, as well as nondriving breaks per working shift.^2^ For example, the current HOS rules limit the total number of working hours per shift to 14 hours and total driving hours to 11 hours. In addition, drivers should take a 34‐hour off‐duty period (with two 1:00 AM to 5:00 AM periods) after 60 on‐duty driving hours in 7 consecutive days or 70 hours in 8 consecutive days. The HOS rules are constantly revised with the advancement in traffic safety research.

Substantial research has been conducted to evaluate truck driver schedules, sleeping, and safety performance. Prolonged working and driving hours within a shift could lead to driving fatigue, which is a major driving risk factor.^3^ Total sleeping time prior to a working shift has been shown to have a direct impact on driving risk.^4^, ^5^ Besides the total sleep duration, other characteristics, such as sleep in the early stage of the non‐work period, and less sleep between 1:00 AM and 5:00 AM, also influence driving risk.^6^ Blanco et al^7^ evaluated the change in driving risk over the 11‐hour on‐duty driving period using a large‐scale naturalistic driving study (NDS) and showed that the risk in the 11th hour is significantly higher than in the first 2 hours of driving. The study also found that safety critical events (SCEs) increased over the 14‐hour working period. Using a different data source and study design, Chen and Xie^8^ confirmed elevated crash risk at the 11th hour of driving. Driver adaption and countermeasures could mitigate adverse effects of fatigue, including sleeping more prior to a long trip, taking rest breaks, and consuming caffeine. For example, rest breaks during long driving session could reduce driving risk.^7^

Modeling the truck driving data is challenging due to the complex nature of driving behaviors and safety events. Various types of SCEs, on‐duty and off‐duty breaks, and nonworking periods occur within a driving shift. Driving and rest behaviors have both direct and indirect effects on the risk of SCEs. In addition, the effect of a covariate may not be constant over time. For instance, the impact of off‐duty sleeping time before a driving task might change over a driving shift due to increasing fatigue over time.^4^ Thus, recurrent event models with time‐varying coefficients are preferred to model complex transportation data.^9^, ^10^ The Bayesian joint modeling approach for multitype recurrent event data has been developed in the recent literature^9^, ^11^, ^12^ due to its flexibility to handle the complicated data structures.

Model assessment and diagnosis are the two important components in Bayesian inference. Commonly used approaches, such as the deviance information criterion (DIC), the conditional predictive ordinate (CPO), and the logarithm of the pseudo‐marginal likelihood (LPML) have been applied to Bayesian recurrent event models.^12^, ^13^, ^14^ However, high dimensionality of the random effects in the multitype recurrent event models often leads to a high cost in computing model assessment criteria.^15^ There is a need to develop reliable and computationally feasible model assessment tools for Bayesian multitype recurrent event models. The discrimination ability is another essential aspect to assess the model fit. A frequently used approach, especially popular in survival analysis, is the concordance index (C‐index).^16^, ^17^, ^18^ However, discrimination ability is not broadly discussed for recurrent event models. To the best of our knowledge, only Kim et al^19^ developed a C‐index for the proportional rate/mean model for recurrent event data within the frequentist framework, which is estimated by the proportion of pairs where the risk prediction scores and the observed event counts are concordant.

In this paper, we jointly model the incidence of SCEs and on‐duty breaks using a Bayesian time‐varying coefficient model for multitype recurrent event data to account for the dependent structure between SCEs and on‐duty breaks. In order to investigate time‐variant driving behaviors such as driving between 1:00 AM and 5:00 AM, we extend the model proposed by Liu and Guo^9^ to incorporate time‐dependent covariates. For Bayesian multitype recurrent event models, we introduce the type‐specific DIC to assess the goodness‐of‐fit of the model to the data for each recurrent event type. The proposed type‐specific DICs are attractive in the sense that (i) the sum of the type‐specific DICs reduces to the overall DIC, which can be used to evaluate the model fit for the whole data, and (ii) they are easily computed using readily available Markov chain Monte Carlo (MCMC) samples from the joint posterior distribution without resorting any analytically intractable integrals. We also consider the LPML for model comparison since it is based on the predictive distribution of the recurrent event data after integrating out of the random effects, which captures the predictive aspect of the model. In addition, an easy‐to‐implement algorithm of the Monte Carlo method based on the CPO Identity II^15^ is developed for computing the LPML for the complex multitype recurrent event models. Three types of C‐indices, within‐event C‐index, between‐event C‐index, and overall C‐index are proposed to quantify discrimination abilities of Bayesian multitype recurrent event models. Each proposed C‐index is a function of the model parameters including the random effects, which is computed at each MCMC sample. Thus, the posterior distributions of the C‐indices are easily obtained as a byproduct of MCMC sampling.

The remainder of the paper is organized as follows. Section 2 introduces the data structure of the commercial truck driver NDS study, the types of recurrent events, and the potentially useful time‐independent and time‐varying predictors. The formulation of the Bayesian multitype recurrent event model with time‐varying coefficients and time‐dependent covariates, the prior specification, and the resulting posterior are given in Section 3. Section 4 presents the detailed development of the Bayesian model assessment criteria and corresponding computational algorithm. In the same section, we propose three types of C‐indices for the Bayesian multitype recurrent event models. Simulation studies are presented in Section 5 and an in‐depth analysis of the NDS data is carried out in Section 6. Concluding remarks and discussions are provided in Section 7.

## 2 COMMERCIAL TRUCK DRIVER NDS

The commercial truck driver NDS is a large‐scale prospective cohort study with the primary goal of evaluating truck driver safety.^7^ A total of 100 commercial truck drivers each drove an instrumented truck for a 4‐week period (four drivers were excluded due to missing data issues). Each truck was instrumented with an advanced data acquisition system. The system collected dozens of key driving parameters, and included multichannel cameras, three‐dimensional accelerometers, GPS, radar, etc. The continuous driving data were collected from ignition‐on to ignition‐off at high frequency (eg 10 Hz for videos and accelerometers). The objectively collected NDS data have provided opportunities to evaluate various risk factors, particularly driver behavior factor due to the comprehensive information collected.^20^

In addition to the driving data, the study also included a detailed daily activity register. The data include 15 on‐duty and off‐duty tasks, such as driving, working, eating, sleep, leisure activities, etc. The participants were asked to record the time of each activity. The activity register data were compared with the objectively recorded driving data to create a set of hybrid activity data containing the metrics of the register data's completeness and the driving data's accuracy.

The data for this study were comprised of 1848 on‐duty driving shifts from the 96 drivers, among whom 74 (77.1%) worked primarily on long‐haul tasks and 21 (22.9%) were line‐haul drivers. There were 91 (94.8%) male and 5 (5.2%) female drivers with an average age of 44 years, ranging from 21 to 73 years. Drivers' experience in driving commercial vehicles varied from 4 weeks to 54 years. A maximum of 11 hours of driving time or a maximum of 14 hours total on‐duty time for each driving shift were observed due to the HOS guidelines set by the Federal Motor Carrier Safety Administration.

### 2.1 Multitype recurrent events

**FIGURE 1:**
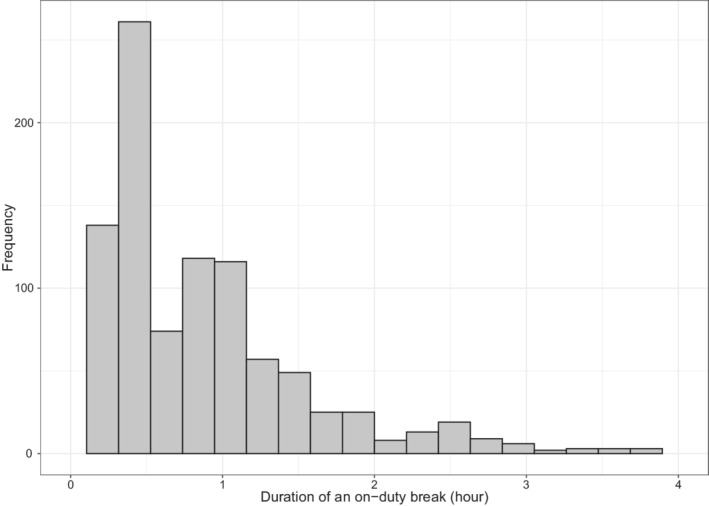
Distribution of the duration of on‐duty breaks

The SCEs and on‐duty breaks were considered as recurrent events that took place during and up to 11 driving hours for each driving shift. The NDS study collected four types of SCEs from the continuous driving data, namely, unintentional lane deviations (ULD), crashes, near‐crashes, and crash‐relevant conflict (CRC) events. These events were identified by an initial screen of kinematic signatures and were confirmed by video recordings. Due to the small number of crash and near‐crash events, these were grouped with CRC events to avoid rare event issues.^9^ As shown in Figure 1, the duration of on‐duty breaks had a median length of 45 minutes with a range from 0.01 to 4 hours. Furthermore, the on‐duty breaks were dichotomized into short breaks (SB) and long breaks (LB) based on the median break duration. Thus, two types of SCEs (ULD and CRC) and two types of on‐duty break (SB and LB) events were considered in the real data analysis. In total, we observed 1124 ULD events from 343 shifts, 1112 CRC events from 548 shifts, 474 SB events from 383 shifts and 436 LB events from 367 shifts.

**FIGURE 2:**
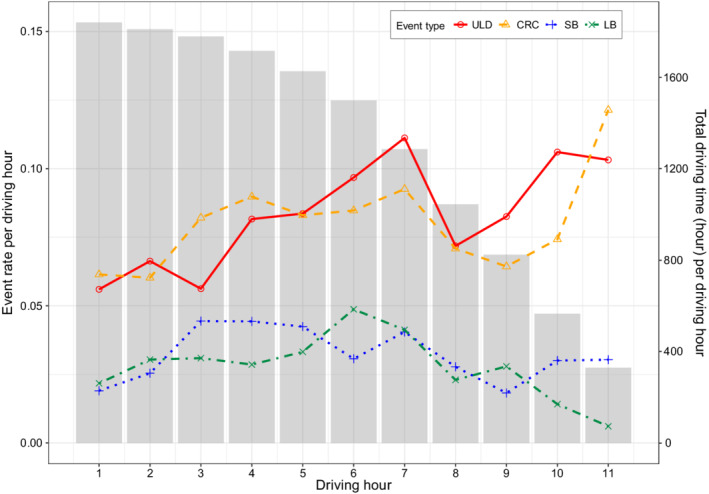
Incidence rate of safety critical events and on‐duty breaks at the first to 11th driving hour

The incidence rates of the four types of recurrent events at each driving hour are displayed in Figure 2. The SCE rates were generally higher than the on‐duty break rates. Both ULD and CRC event rates climbed from the start of driving shifts to the seventh driving hour. After a slight drop, the SCE rates increased again at the last two driving hours. The event rate curves for the on‐duty breaks were more flat compared to the SCEs. Particularly, the LB event rate reached its highest at the sixth hour and decreased in the last several driving hours. This likely indicates that drivers might not take a LB toward the later driving hours since this would be close to the end of a driving shift.

### 2.2 Time‐independent variables

One of the primary interests is to investigate if taking a compensatory long off‐duty rest after an intensive on‐duty period helps commercial truck drivers improve their driving performance. In the NDS data, we identified whether drivers needed a 34‐hour off‐duty break before a driving shift by checking to see if they drove for at least 60 hours in a 7‐day rolling window or at least 70 hours in an 8‐day rolling window before the end of the previous driving shift. If a driver did not meet the criterion, we considered the corresponding driving shift to be “no need to restart." Otherwise, the corresponding driving shift was grouped to “restarting driving" or “not restarting driving" based on if the driver took an off‐duty break for at least 34 hours. The 1848 driving shifts contained 1323 (71.6%) “no need to restart" shifts, 80 (4.3%) “restarting driving" shifts and 445 (24.1%) “not restarting driving" shifts. For the 525 driving shifts after a long concentrated working period, the drivers in 84.8% of those shifts did not take an off‐duty break for at least 34 hours, which indicates a low compliance rate for this HOS recommendation.

The off‐duty sleeping time, the number of nondriving tasks, the shift starting time, and the driver type were also considered as time‐independent predictors for influencing the incidence rates of SCEs and on‐duty breaks. The off‐duty sleeping time before a driving shift was categorized into three groups: normal sleeping (7‐9 hours), insufficient sleeping (less than 7 hours), and abundant sleeping (more than 9 hours). This study consisted of 1112 (60.2%) shifts in the normal sleeping group, 271 (14.7%) shifts in the insufficient sleeping group, and 465 (25.2%) shifts in the abundant sleeping group. It has been shown that the off‐duty sleeping groups have different SCE risk patterns over the 11‐hour driving time.^9^, ^10^ The number of nondriving tasks had a mean of 2.86 (SD = 2.08) and a range of frequency from 0 to 13. We considered three shift starting time groups: 667 (36.1%) shifts started in the morning (5:00 AM‐9:00 AM), 768 (41.6%) shifts started in the daytime (9:00 AM‐8:00 PM), and 413 (22.3%) shifts started at night (8:00 PM‐5:00 AM).

Table 1 presents the descriptive statistics of the driving behaviors across the three restarting groups. The drivers in the “not restarting driving" group reported a higher chance of getting abundant off‐duty sleep before a driving shift, and a lower percentage of starting driving at night compared to the other two groups. This shows that the drivers who did not take a long off‐duty break to recover from an intensive driving period may need a longer off‐duty sleep duration to compensate for resting and avoid tiring late‐night driving shift. In addition, most of the driving shifts (99.2%) following with a long consecutive on‐duty period were from the long‐haul drivers, which explains that those shifts had a longer shift duration and fewer number of nondriving tasks.

**TABLE 1:** Descriptive table of shift‐level characteristics in the three restarting groups

Shift‐level characteristics		No need to restart	Restarting driving	Not restarting driving	*P*‐value^*a*^
		Mean(SD)			
Length of shift		7.61 (2.52)	8.37 (2.47)	8.05 (2.69)	<0.001
Number of nondriving work		3.04 (2.19)	2.85 (1.93)	2.33 (1.65)	<0.001
		Frequency (Proportion)			
Sleeping Group	Insufficient	235 (17.8%)	10 (12.5%)	26 (5.8%)	<0.001
	Normal	782 (59.1%)	50 (62.5%)	280 (62.9%)	
	Abundant	306 (23.1%)	20 (25.0%)	139 (31.2%)	
Starting Time	Morning	435 (32.9%)	25 (31.2%)	207 (46.5%)	<0.001
	Daytime	546 (41.3%)	38 (47.5%)	184 (41.3%)	
	Night	342 (25.9%)	17 (21.2%)	54 (12.1%)	
Driver Type	Line‐haul	399 (30.2%)	3 (3.8%)	1 (0.2%)	<0.001
	Long‐haul	924 (69.8%)	77 (96.3%)	444 (99.8%)	

^*a*^ *P*‐values are from the Kruskal‐Wallis test for continuous variables and the Pearson Chi‐square/Fisher's exact test for categorical variables.

### 2.3 Time‐dependent variables

The local times of the driving activities and the occurrence of any events were also collected in the NDS data. Driving at 1:00 AM to 5:00 AM is coded as a binary time‐dependent covariate in the 11 driving hours. Among all the observed driving shifts, 547 (29.6%) shifts overlapped with 1:00 AM to 5:00 AM, with an average late‐night driving time of 2.54 hours per driving shift.

## 3 BAYESIAN MODELING AND ESTIMATION OF MULTITYPE RECURRENT EVENT DATA

### 3.1 Model specification

We jointly model the intensity functions of different types of SCEs and on‐duty break events using a multitype Poisson process model with both fixed and time‐varying coefficients following Liu and Guo.^9^ For the i th driving shift, i=1,…,n, let $X_{i}={\left(x_{i1},\ldots x_{ip}\right)}^{\prime }$, $Z_{i}={\left(z_{i1},\ldots ,z_{iq}\right)}^{\prime }$, and $W_{i}(t)={\left(w_{i1}(t),\ldots ,w_{is}(t)\right)}^{\prime }$ represent the shift‐level predictors with fixed effect, shift‐level predictors with time‐varying effect, and time‐dependent covariates at time t, respectively. Conditioning on the random effect $b_{ij}$, we assume that the intensity function for the i th driving shift and the j th event type is given by

(1) $$\lambda_{ij}(t|X_{i},W_{i}(t),Z_{i},b_{ij})=\exp\{\alpha_{j}^{\prime }X_{i}+\gamma_{j}^{\prime }W_{i}(t)+\beta_{0j}(t)+z_{i1}\beta_{1j}(t)+\cdots +z_{iq}\beta_{qj}(t)+b_{ij}\},$$

where $\alpha_{j}$ and $\gamma_{j}$ are two vectors of the linear regression coefficients for the time‐independent and time‐dependent covariates, respectively; $\beta_{0j}(t)$ is a smooth function for the log baseline intensity of the j th event type; $\beta_{1j}(t)\ldots \beta_{qj}(t)$ are the time‐varying coefficients of the covariates in $Z_{i}$; $b_{ij}$ is a shift‐level random effect for j=1,…,J. We further assume that $b_{i}={\left(b_{i1},\ldots ,b_{iJ}\right)}^{\prime }$ follows a multivariate normal distribution with mean ${\left(0,\ldots ,0\right)}^{\prime }$ and covariance ∑. By incorporating the log‐normal frailties with the dependence structure ∑, the model is able to capture the variability within each type of the recurrent events and the association between two types of events.

The baseline intensity function and the time‐varying coefficients, $\beta_{lj}(t)$, l=0,…q, j=1,…,J, are unknown smooth functions, which are modeled by polynomial splines with equally spaced knots on the 11 driving hours. We adopt the B‐splines with degree of ν, which are widely used in recurrent event models with nonparametric intensity functions.^9^, ^10^, ^12^ Let $h_{l}$ denote the number of knots and $B_{lh}(t)$ represent the value of the h th B‐spline at t, where $h=1,\ldots ,H_{l}$ and $H_{l}=h_{l}+\nu$. Then, the smooth function $\beta_{lj}(t)$ is expressed by a linear combination of the B‐spline basis functions $B_{lh}(t)$'s given by $\beta_{lj}(t)=\sum_{h=1}^{H_{l}}B_{lh}(t)\beta_{hlj}=B_{l}^{\prime }(t)\beta_{lj}$, where $B_{l}(t)={\left(B_{l1}(t),\ldots ,B_{lH_{l}}(t)\right)}^{\prime }$ and $\beta_{lj}={\left(\beta_{1lj},\ldots ,\beta_{H_{l}lj}\right)}^{\prime }$ is a vector of the corresponding regression coefficients.

For the i th driving shift, let $t_{ijk}$ be the event time of the k th type j event, let $n_{ij}$ denote the number of type j events occurring before the follow‐up time $c_{i}$, and $D_{i}=\{X_{i},Z_{i},W_{i},c_{i}\}$ stands for the observed shift‐level data. Let $t_{ij}=\{t_{ij1},\ldots ,t_{ijn_{ij}}\}$ and $\Omega_{j}=\{\alpha_{j},\gamma_{j},\beta_{lj},l=0,\ldots ,q\}$ represent the sets of the j th type‐specific event time and parameters, respectively. Write $t_{i}=\{t_{i1},\ldots ,t_{iJ}\}$ and $\Omega =\{\Omega_{1},\ldots ,\Omega_{J}\}$. The conditional likelihood given the random effects $b_{i}$ for the i th driving shift is written as

(2) $$L_{i}(\Omega |b_{i},t_{i},D_{i})=\overset{J}{\underset{j=1}{\prod }}L_{ij}(\Omega_{j}|b_{ij},t_{ij},D_{i}),$$

where the type‐specific conditional likelihood is defined as

(3) $$L_{ij}(\Omega_{j}|b_{ij},t_{ij},D_{i})=\overset{n_{ij}}{\underset{k=1}{\prod }}\lambda_{ij}(t_{ijk}|X_{i},W_{i}(t_{ijk}),Z_{i},b_{ij})\exp(-\Lambda_{ij}(c_{i})),$$

where $\Lambda_{ij}(c_{i})=\int_{0}^{c_{i}}\lambda_{ij}(t|X_{i},W_{i}(t),Z_{i},b_{ij})dt$ and $\lambda_{ij}(t_{ijk}|X_{i},W_{i}(t_{ijk}),Z_{i},b_{ij})$ is defined in (1). We note that $\Lambda_{ij}(c_{i})$ does not have a closed form due to the time‐varying coefficients, so we adopt a trapezoid method for obtaining an approximation of $\Lambda_{ij}(c_{i})$ following Liu and Guo.^9^

### 3.2 Prior and posterior

Controlling the smoothness of the spline function is crucial in the time‐varying coefficient models. The smoothness is essentially controlled by the number of knots of basis splines and the order of penalty of smoothing. To allow for a flexible fit, the number of knots should be large enough, which may introduce an over‐fitting problem. The penalized B‐spline (P‐spline), which uses a relatively large number of knots and a penalty of smoothing on the objective function to control the smoothness of the fit, has been widely studied in the literature.^21^, ^22^ Following Liu and Guo^9^ and Lang and Brezger,^22^ We specify the shrinkage priors for the coefficients of the B‐splines given by

(4) $$\beta_{lj}|\tau_{lj}\propto \exp\left\{-\frac{\tau_{lj}}{2}\beta_{lj}^{\prime }(D_{l}^{\prime }D_{l})\beta_{lj}\right\},$$

where $D_{l}$ is a proper matrix such that $D_{l}\beta_{lj}={\left(\Delta^{r}\beta_{r+1,lj},\ldots ,\Delta^{r}\beta_{K_{l},lj}\right)}^{\prime }$ with rank $K_{l}-r$. Δ denotes the difference between the coefficients of adjacent B‐splines and r denote a finite order of difference. For instance, the first and second order differences of $\beta_{hlj}$ are given by $\Delta \beta_{hlj}=\beta_{hlj}-\beta_{h-1,lj}$ and $\Delta^{2}\beta_{hlj}=\Delta \Delta \beta_{hlj}=\beta_{hlj}-2\beta_{h-1,lj}+\beta_{h-2,lj}$, respectively. The roughness penalty parameter $\tau_{lj}$ is considered as random and it is estimated jointly with other unknown parameters. A Gamma prior is assigned to $\tau_{lj}$ such that $\tau_{lj}∼\text{Gamma}(a_{\tau },b_{\tau })$, where $a_{\tau }$ is the shape parameter and $b_{\tau }$ is a small rate parameter of the Gamma distribution. However, the choice of $b_{\tau }$ highly impacts the smoothness of the Bayesian P‐spline.^23^ Thus, we reparameterize this parameter as $b_{\tau }=\delta_{lj}a_{\tau }$ and then assign a Gamma hyperprior to $\delta_{lj}$. Specifically, we specify $\tau_{lj}∼\text{Gamma}(a_{\tau },a_{\tau }\delta_{lj})$ and $\delta_{lj}∼\text{Gamma}(a_{\delta },b_{\delta })$. In Section 5 below, we specify $a_{\tau }=1$, $a_{\delta }=0.01$ and $b_{\delta }=0.01$, which lead to a relatively non informative prior.

Since there is no prior information available for the analysis, a noninformative prior $N(0,10^{3})$ is assigned to each of the time‐independent coefficients $\alpha_{j}$ and $\gamma_{j}$. With $\tilde{\Omega }=\{\Omega ,\sum ,\tau_{lj},\delta_{lj},1\leq j\leq J,0\leq l\leq q\}$ denoting all the parameters and hyperparameters in the model and priors, the joint posterior distribution is written as

(5) $$\begin{array}{ll} p(\tilde{\Omega }|t_{i}D_{i},1\leq i\leq n) & \propto \overset{n}{\underset{i=1}{\prod }}L_{i}(\Omega |b_{i},D_{i})p\left(b_{i}|\sum \right)\\ & \times \overset{J}{\underset{j=1}{\prod }}\overset{q}{\underset{l=0}{\prod }}p\left(\beta_{lj}|\tau_{lj}\right)p\left(\tau_{lj}|\delta_{lj}\right)p\left(\delta_{lj}\right)\overset{J}{\underset{j=1}{\prod }}p\left(\alpha_{j}\right)p\left(\gamma_{j}\right)p(\sum ). \end{array}$$

**FIGURE 3:**
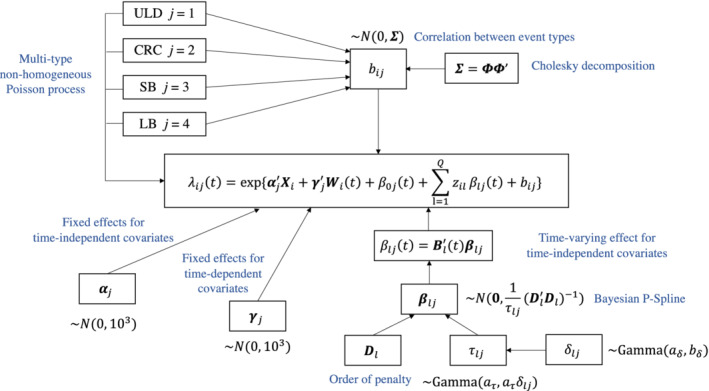
Model diagram of the multitype Poisson process model with prior specifications.

To achieve better convergence of MCMC sampling, the Cholesky decomposition is widely applied in modeling the covariance matrix of the random effects for longitudinal data^24^, ^25^ and multi‐type recurrent event data.^9^, ^12^, ^13^ Let $\sum =\Phi \Phi^{\prime }$, where Φ is a lower triangular matrix with positive diagonal entries $\phi_{jj}$ and unrestricted off‐diagonal entries $\phi_{jk},j>k$. The random effects of the i th driving shift are reparameterized as $b_{i}=\Phi u_{i}$, where $u_{i}∼N_{J}(0,I_{J})$ and $I_{J}$ denotes a J×J identity matrix. We assign the priors to the entries of Φ such that $\phi_{jj}∼N^{+}(0,1000)\;\text{and}\;\phi_{jk}∼N(0,1000)$, where $N^{+}$ denotes the truncated normal distribution defined on $R^{+}=(0,\infty )$. A diagram of the multi‐type Poisson process model with the prior specification is displayed in Figure 3.

To sample the parameters from the joint posterior, we follow Liu and Guo to implement a Metropolis‐adjusted Langevin Algorithm^26^ designed for sampling the penalized spline parameters in the Bayesian multitype Poisson process model.^9^ This algorithm integrated with a joint sampling scheme helps achieve better convergence in the posterior sampling, with addressing the issue of the strong dependence of spline coefficients due to the random walk for the roughness penalty.

## 4 BAYESIAN MODEL ASSESSMENTS

### 4.1 Deviance information criterion

To assess the model fit of the data for the j th type of recurrent events, we specify the type‐specific deviance as

(6) $${\text{Dev}}_{j}(\theta_{j})=-2\overset{n}{\underset{i=1}{\sum }}\log L_{ij}(\theta_{j}|t_{ij},D_{i}),$$

where $\theta_{j}=\{\beta_{j},\alpha_{j},\gamma_{j},b_{ij}\}$ are the model parameters and the random effects for the j th type of event, and the type‐specific likelihood $L_{ij}(\theta_{j}|t_{ij},D_{i})$ is given by (3). Following Reference 27, the type‐specific DIC for the j th event type is defined as

(7) $${\text{DIC}}_{j}={\text{Dev}}_{j}({\bar{\theta }}_{j})+2p_{D[j]},$$

where $p_{D[j]}={\bar{\text{Dev}}}_{j}(\theta_{j})-{\text{Dev}}_{j}({\bar{\theta }}_{j})$ is the effective number of parameters for the j th event type, and ${\bar{\theta }}_{j}$ and ${\bar{\text{Dev}}}_{j}(\theta_{j})$ are the posterior means of $\theta_{j}$ and ${\text{Dev}}_{j}(\theta_{j})$, respectively. Write $\theta =\{\theta_{j},j=1,..,J\}$. The overall DIC for all types of recurrent events is further given by

(8) $$\text{DIC}=\text{Dev}(\bar{\theta })+2p_{D}=\overset{J}{\underset{j=1}{\sum }}{\text{DIC}}_{j},$$

where the overall deviance function and overall effective number of parameters can also be decomposed to the sum of the type‐specific components such that $\text{Dev}(\theta )=\sum_{j=1}^{J}{\text{Dev}}_{j}(\theta_{j})$ and $p_{D}=\sum_{j=1}^{J}p_{D[j]}$.

The type‐specific ${\text{DIC}}_{j}$ in (7) is attractive since it not only avoids analytical intractable integrals by treating the random effects as parameters in the likelihood function, but also provides a useful tool to compare Bayesian recurrent event models for a certain event type. The overall DIC, which is naturally decomposed into the type‐specific ${\text{DIC}}_{j}$'s, measures the model fit of the data from all types of events together.

### 4.2 CPO and LPML

Let $t_{-i}$ and $D_{-i}$ denote the observed data without the i th driving shift, and write Γ={Ω,∑}. The CPO^14^, ^28^, ^29^ for the i th driving shift is defined as

(9) $${\text{CPO}}_{i}=\int f(t_{i}|\Gamma ,D_{i})p(\Gamma |t_{-i},D_{-i})d\Gamma ,$$

where

(10) $$f(t_{i}|\Gamma ,D_{i})=\int L_{i}(\Gamma |b_{i},D_{i})p(b_{i}|\sum )db_{i},$$

and $p(\Gamma |t_{-i},D_{-i})$ denotes the marginal posterior distribution of Γ constructed by the data excluding the i th driving shift. Following the results from Chen et al.^30^ ${\text{CPO}}_{i}$ can be rewritten as (CPO Identity I^15^)

(11) $${\text{CPO}}_{i}=\frac{1}{\int \frac{1}{f(t_{i}|\Gamma ,D_{i})}p(\Gamma |t,D)d\Gamma }.$$

The CPO Identity I leads to a popular Monte Carlo estimate of CPO using the MCMC samples from the posterior distribution.^30^ Let $\Gamma_{m}$ stand for the m th MCMC sample of Γ for m=1,…,M. A Monte Carlo estimate of ${\text{CPO}}_{i}^{-1}$ is given by

(12) $${\hat{\text{CPO}}}_{i}^{-1}=\frac{1}{M}\overset{M}{\underset{m=1}{\sum }}\frac{1}{f(t_{i}|\Gamma_{m},D_{i})}.$$

However, this Monte Carlo estimate involves the integral of the joint likelihood over the random effects $b_{i}$'s, which is computationally prohibitive under the complex multitype recurrent event models with time‐varying effects and time‐dependent covariates. Thus, we develop an easy‐to‐implement algorithm to approximate ${\text{CPO}}_{i}$, which directly uses the MCMC samples generated from the joint posterior distribution (5).

Let $w_{i}(b_{i})$ be a normalized weight function such that $\int w_{i}(b_{i})db_{i}=1$ and let $\left\{(\Gamma_{m},b_{im}),m=1,\ldots ,M\right\}$ denote the MCMC samples of ($\Gamma ,b_{i}$) after burn‐in. Following the CPO Identity II,^15^ a Monte Carlo estimate of the shift‐level CPO is given by

(13) $${\hat{\text{CPO}}}_{i}^{-1}=\frac{1}{M}\overset{M}{\underset{m=1}{\sum }}\frac{w_{i}(b_{im})}{f(t_{i},b_{im}|\Gamma_{m},D_{i})}.$$

The normalized weight function $w_{i}(b_{i})$ can be constructed by a multivariate normal density via the Laplace approximation of the joint density given by

(14) $$f(t_{i},b_{i}|\Gamma ,D_{i})=\overset{J}{\underset{j=1}{\prod }}\left\{\overset{n_{ij}}{\underset{k=1}{\prod }}\lambda_{ij}(t_{ijk}|D_{i},b_{ij})\exp(-\Lambda_{ij}(c_{i}))\right\}p(b_{i}).$$

After some algebra, the weight $w_{i}$ is set as

(15) $$w_{i}(b_{i})=\phi (b_{i};\tilde{\mu },\tilde{\sum }),$$

where

$$\tilde{\mu }=b_{i0},\tilde{\sum }={\left(-\frac{\partial^{2}h(b_{i0})}{\partial b_{i}\partial b_{i}^{T}}\right)}^{-1},$$

$$h(b_{i})=\overset{J}{\underset{j=1}{\sum }}n_{ij}b_{ij}-\overset{J}{\underset{j=1}{\sum }}{\tilde{\Lambda }}_{ij}(c_{i})\exp(b_{ij})-\frac{1}{2}b_{i}^{T}\sum^{-1}b_{i},$$

ϕ is the normal density function with the mean vector $\tilde{\mu }$ and the covariance matrix $\tilde{\sum }$, and $b_{i0}$ is the mode of $h(b_{i})$ which can be calculated by numerically solving $\frac{\partial h(b_{i})}{\partial b_{i}}=0$. The detailed derivation of the weight function $w_{i}(b_{i})$ is given in Appendix. Therefore, all the components in the Monte Carlo estimate (13) are able to be directly computed by the MCMC samples from the joint posterior distribution. Furthermore, the LPML^31^ is defined as

$$\text{LPML}=\overset{n}{\underset{i=1}{\sum }}\log({\text{CPO}}_{i}).$$

A larger LPML suggests a better model fit.

### 4.3 C‐index for multitype recurrent event model

#### 4.3.1 Within‐event C‐index

Let $N_{ij}(t)$ denote the number of type j events occurring on the time interval [0,t] of the i th driving shift. Conditioning on the random effects, the expectation of the number of type j events at time t for the i th driving shift is given by

(16) $$\mu_{ij}(t|b_{ij})=E[N{\left(t\right)}_{ij}|b_{ij}]=\int_{0}^{t}\lambda_{ij}(u|X_{i},W_{i}(u),Z_{i},b_{ij})du,$$

where the conditional intensity function $\lambda_{ij}(u|X_{i},W_{i}(u),Z_{i},b_{ij})du$ is given in (1). We rewrite $\lambda_{ij}(u|X_{i},W_{i}(u),Z_{i},b_{ij})du=\exp(b_{ij})\lambda_{ij}(u|X_{i},W_{i}(u),Z_{i})$. The expected number of type j events at time t for the i th driving shift is given by

(17) $$\begin{array}{ll} \mu_{ij}(t)=E_{b_{i}}[\mu_{ij}(t|b_{ij})] & =\int_{b}\int_{0}^{t}\exp(b_{ij})\lambda_{ij}(u|X_{i},W_{i}(u),Z_{i})du\;p(b_{i}|\sum )db_{i}\\ & =\int_{0}^{t}\lambda_{ij}(u|X_{i},W_{i}(u),Z_{i})du\;\int_{-\infty }^{\infty }\exp(b_{ij})p(b_{ij}|\sigma_{jj}^{2})db_{ij}\\ & =\exp(\frac{\sigma_{jj}^{2}}{2})\int_{0}^{t}\lambda_{ij}(u|X_{i},W_{i}(u),Z_{i})du, \end{array}$$

where $\sigma_{jj}^{2}$ is the j th diagonal entry of the covariance matrix ∑.

To measure the model's discrimination ability for the type j event, we propose a within‐event C‐index based on the comparable pairs constructed by two driving shifts with different observed event rates of the type j event. The within‐event C‐index for event type j is defined as

(18) $$C_{W,j}=P\left(\frac{\mu_{ij}(c_{i})}{c_{i}}>\frac{\mu_{i^{\prime }j}(c_{i^{\prime }})}{c_{i^{\prime }}}|\frac{N{\left(c_{i}\right)}_{ij}}{c_{i}}>\frac{N{\left(c_{i^{\prime }}\right)}_{i^{\prime }j}}{c_{i^{\prime }}}\right),$$

where $i\neq i^{\prime }$, i=1,…,n, $i^{\prime }=1,\ldots ,n$, and the follow‐up time $c_{i}$ is independent to the occurrence of the type j events. A comparable pair is concordant if the comparisons between the expected event rates and between the observed event rates are consistent. Then, we estimate $C_{W,j}$ by the proportion of concordant pairs among all the comparable pairs, which is given by

(19) $${\hat{C}}_{W,j}=\frac{\overset{n}{\underset{i=1}{\sum }}\overset{n}{\underset{i^{\prime }=1}{\sum }}I\left\{\frac{N{\left(c_{i}\right)}_{ij}}{c_{i}}>\frac{N{\left(c_{i^{\prime }}\right)}_{i^{\prime }j}}{c_{i^{\prime }}}\right\}I\left\{\frac{\mu_{ij}(c_{i})}{c_{i}}>\frac{\mu_{i^{\prime }j}(c_{i^{\prime }})}{c_{i^{\prime }}}\right\}}{\overset{n}{\underset{i=1}{\sum }}\overset{n}{\underset{i^{\prime }=1}{\sum }}I\left\{\frac{N{\left(c_{i}\right)}_{ij}}{c_{i}}>\frac{N{\left(c_{i^{\prime }}\right)}_{i^{\prime }j}}{c_{i^{\prime }}}\right\}}.$$

#### 4.3.2 Between‐event C‐index

For the multitype recurrent event model, comparable pairs can also be constructed between two types of events since the event rates are comparable across different event types. For two different types of recurrent event $j\neq j^{\prime }$, the between‐event C‐index is defined as

(20) $$C_{B,jj^{\prime }}=P\left(\frac{\mu_{ij}(c_{i})}{c_{i}}>\frac{\mu_{i^{\prime }j^{\prime }}(c_{i^{\prime }})}{c_{i^{\prime }}}|\frac{N{\left(c_{i}\right)}_{ij}}{c_{i}}>\frac{N{\left(c_{i^{\prime }}\right)}_{i^{\prime }j^{\prime }}}{c_{i^{\prime }}}\right),$$

Similar to the within‐event C‐index, an estimate of the C‐index between the types j and $j^{\prime }$ events is given by

(21) $${\hat{C}}_{B,jj^{\prime }}=\frac{\overset{n}{\underset{i=1}{\sum }}\overset{n}{\underset{i^{\prime }=1}{\sum }}I\left\{\frac{N{\left(c_{i}\right)}_{ij}}{c_{i}}>\frac{N{\left(c_{i^{\prime }}\right)}_{i^{\prime }j^{\prime }}}{c_{i^{\prime }}}\right\}I\left\{\frac{\mu_{ij}(c_{i})}{c_{i}}>\frac{\mu_{i^{\prime }j^{\prime }}(c_{i^{\prime }})}{c_{i^{\prime }}}\right\}}{\overset{n}{\underset{i=1}{\sum }}\overset{n}{\underset{i^{\prime }=1}{\sum }}I\left\{\frac{N{\left(c_{i}\right)}_{ij}}{c_{i}}>\frac{N{\left(c_{i^{\prime }}\right)}_{i^{\prime }j^{\prime }}}{c_{i^{\prime }}}\right\}},$$

where i=1,…,n and $i^{\prime }=1,\ldots ,n$. The between‐event C‐index can be interpreted as the proportion of concordant pairs among all the comparable pairs with event rates from two different event types. It evaluates the model's discrimination ability between two different event types.

#### 4.3.3 Overall C‐index

Generally considering two event types, there are only two ways to construct the comparable pairs, either within one certain event type or between two different event types. Therefore, without loss of generality, we propose the overall C‐index for a recurrent event model with a total of two types of events, which is given by

$$C_{O}=\pi_{W,1}C_{W,1}+\pi_{W,2}C_{W,2}+\pi_{B,12}C_{B,12},$$

where $\pi_{W,1}$($\pi_{W,2}$) and $\pi_{B,12}$ denote the probability of a within‐event comparable pair from the type 1(2) event and the probability of a between‐event comparable pair constructed by the two types of events, respectively. The overall C‐index with more than two types of events can be decomposed similarly with J within‐event C‐indices and $\left(\begin{array}{c} J\\ 2 \end{array}\right)$ between‐event C‐indices. The overall C‐index measures the model's discrimination performance on all types of recurrent events simultaneously.

Within the Bayesian framework, each of the proposed C‐indices is a function of the model parameters. Instead of a single value of a C‐index within the frequentist framework, we can obtain the posterior distribution of the C‐index by the posterior samples of the model parameters. Thus, the posterior values of the within‐event C‐index, the between‐event C‐index, and the overall C‐index can be calculated by the MCMC samples from the joint posterior, denoted by ${\hat{C}}_{W,j}^{\left(m\right)}$, ${\hat{C}}_{B,jj^{\prime }}^{\left(m\right)}$, and ${\hat{C}}_{O}^{\left(m\right)}$, respectively, where m=1,…,M and M is the iteration of MCMC chain. The means of ${\hat{C}}_{W,j}^{\left(m\right)}$, ${\hat{C}}_{B,jj^{\prime }}^{\left(m\right)}$, and ${\hat{C}}_{O}^{\left(m\right)}$ are considered as the point estimates of the C‐indices. In addition, the 95% credible interval of each C‐index can also be easily obtained based on the posterior values.

## 5 SIMULATION STUDIES

### 5.1 Simulation study 1: DIC and LPML

We conduct a simulation study to evaluate the empirical performance of the proposed type‐specific DIC, overall DIC and LPML on model comparison. We generate 100 datasets with multitype recurrent event data from multitype nonhomogeneous Poisson process models using the R package "reda." Specifically, in each simulation, we simulate 500 driving shifts with two types of recurrent event data and a fixed follow‐up time $c_{i}=10$. For each of the driving shifts, we firstly simulate the random effects $b_{i}$ from a bivariate normal distribution $N_{2}(0,\sum )$ where $\sum =\left(\begin{array}{cc} 2 & 1\\ 1 & 2 \end{array}\right)$. Three shift‐level covariates, $X_{i}$, $Z_{i1}$, and $Z_{i2}$, are generated independently from Bernoulli(0.5), where $X_{i}$ has time‐independent effects and $Z_{i1}$ and $Z_{i2}$ have time‐varying effects on the intensities. Conditional on the simulated random effects and the covariates, we then simulate the type j events from a nonhomogeneous Poisson process model with the type‐specific conditional intensity function given by

(22) $$\lambda_{ij}(t|X_{i},Z_{i1},Z_{i2},b_{ij})=\exp\{\alpha_{j}X_{i1}+\beta_{0j}(t)+Z_{i1}\beta_{1j}(t)+Z_{i2}\beta_{2j}(t)+b_{ij}\},$$

where $\alpha_{1}=\alpha_{2}=0.6$ and each $\beta_{lj}(t)$ is defined by a linear combination of the cubic B‐spline basis functions $\beta_{lj}(t)=\sum_{k=1}^{K+3}B_{k}(t)\beta_{ljk}$ where l=0,1,2, K=6, and j=1,2. The knots are uniformly placed on the time axis. The specific settings of $\beta_{ljk}^{\prime }s$ and the true curves of $\beta_{lj}{\left(t\right)}^{\prime }s$ are given in Figure S1. We consider the following six scenarios to fit different models to each of the simulated datasets:

(1) TRUE: Fit the model (22) with the true number (K=6) of knots of the P‐spline for the baseline intensity functions and the time‐varying coefficients.
(2) MoreK10: Fit the model (22) with 10 knots (K=10) of the P‐spline basis for the baseline intensity functions and the time‐varying coefficients.
(3) LessK3: Fit the model (22) with 3 knots (K=3) of the P‐spline basis for the baseline intensity functions and the time‐varying coefficients.
(4) MissZ2: Fit a misspecified model with the true number (K=6) of knots, but without the predictor $Z_{i2}$ with time‐varying effects.
(5) MissX: Fit a misspecified model with the true number (K=6) of the knots, but without the predictor $X_{i}$ with fixed effects.
(6) MissXZ2: Fit a misspecified model with the true number (K=6) of the knots, but without both $X_{i}$ and $Z_{i2}$.

For each of the simulated dataset, we take 3000 MCMC samples after 2000 burn‐in iterations for the model in each of the scenarios. A sensitivity analysis in regards to the choice of the hyperparameter $a_{\tau }$ is conducted under the TRUE model. No differences in coverage probability of the baseline intensity and time‐varying coefficients are observed among TRUE models with different choices of $a_{\tau }$ (Figure S2). The type‐specific DIC, the overall DIC, and the LPML are calculated for each of the models in each simulation.

**FIGURE 4:**
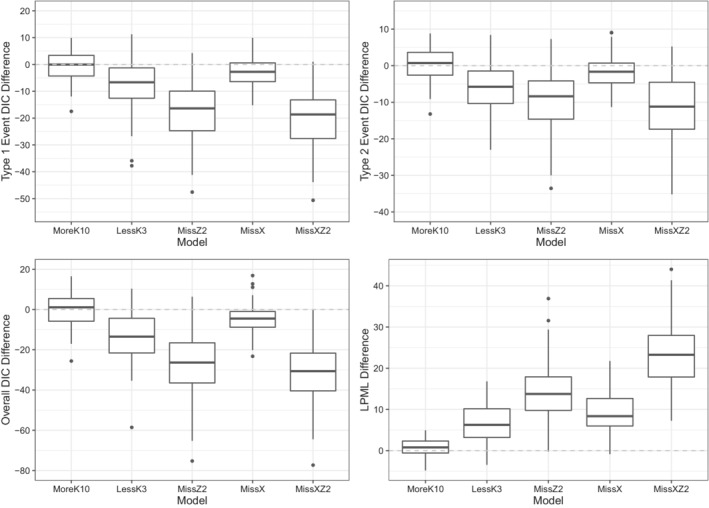
Boxplots of the type‐specific deviance information criterion (DIC) difference, overall DIC difference, and logarithm of the pseudo‐marginal likelihood difference between TRUE and each of MoreK10, LessK3, MissZ2, MissX, and MissXZ2

Figure 4 shows the boxplots of the type‐specific DIC difference, the overall DIC difference, and the LPML difference between TRUE and each of the misspecified models. The summary statistics of DIC and LPML differences are given in Table S1. A negative DIC difference or a positive LPML difference suggests a better fit of TRUE in each of the comparisons. We see that except for MoreK10, the boxplots of the type‐specific DIC difference and the overall DIC difference are almost below 0, and the boxplots for the LPML difference are almost above 0. This indicates that the proposed DIC and LPML are effective in distinguish the better model in both type‐specific and overall perspective. In addition, LPML (LPML difference median = 0.776, IQR = (−0.576, 2.336), % of >0 = 67%) performs slightly better than the overall DIC (Overall DIC difference median = 1.102, IQR = (−5.787, 5.477), % of <0 = 44%) when comparing MoreK10 with TRUE.

### 5.2 Simulation study 2: C‐index

We perform a simulation study to examine the empirical performance on assessing the discrimination ability of the proposed C‐indices. Similar to Simulation Study 1, we generate 100 datasets based on the random effects $b_{i}∼N_{2}(0,\sum )$ where $\sum =\left(\begin{array}{cc} 2 & 0.707\\ 0.707 & 1 \end{array}\right)$ and the conditional intensity function given by

(23) $$\lambda_{ij}(t|X_{i1},X_{i2},Z_{i1},b_{ij})=\exp\{\alpha_{1j}X_{i1}+\alpha_{2j}X_{i2}+\beta_{0j}(t)+Z_{i1}\beta_{1j}(t)+b_{ij}\},$$

where $\alpha_{11}=1.5,\alpha_{12}=0.75,\alpha_{21}=0.75,\alpha_{22}=1.5$, and $X_{i1}$ and $X_{i2}$ are independently sampled from N(0,1). The baseline intensity function and time‐varying coefficients $\beta_{lj}(t)$ have the same settings as those in Simulation Study 1. For each of the simulated data, we fit the following four models with the true number and location of knots:

(1) TRUE: The true model (22) with the correct specification of the covariates.
(2) MissX1: Fit a misspecified model without the covariate $X_{i1}$.
(3) MissX2: Fit a misspecified model without the covariate $X_{i2}$.
(4) MissX1X2: Fit a misspecified model without both covariates $X_{i1}$ and $X_{i2}$.

Same as Simulation Study 1, we take 3000 MCMC samples after 2000 burn‐in iterations for each of the models. The posterior means of the within‐event C‐index, the between‐event C‐index, and the overall C‐index are calculated for each of the models.

**FIGURE 5:**
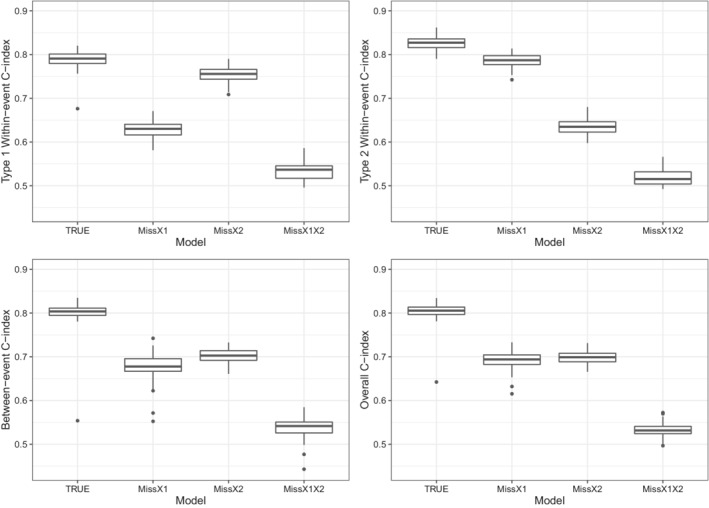
Boxplots of the within‐event C‐index, between‐event C‐index, and overall C‐index of TRUE, MissX1, MissX2, and MissX1X2

Figure 5 displays the boxplots of the posterior means of the three types of C‐indices. The summary statistics of the C‐index simulation results are given in Table S2. It shows that the proposed C‐indices correctly indicate the discriminative abilities of the models, where TRUE obtains the highest discriminative ability and MissX1X2 has the lowest discriminative ability in all types of the C‐indices. The within‐event C‐index also correctly reflects the effect size of the missing predictors. Specifically, we see that MissX1 has a lower type 1 within‐event C‐index (Median = 0.630, IQR = (0.616, 0.641)) compared with MissX2 (Median = 0.756, IQR = (0.744, 0.766)) since to the larger effect of $X_{i1}$ ($\alpha_{11}=1.5$) on the intensity function of the type 1 event than $X_{i2}$ (($\alpha_{21}=0.75$)). Similarly, MissX1 has a higher type 2 within‐event C‐index (Median = 0.787, IQR = (0.777, 0.797)) than MissX2 (Median = 0.635, IQR = (0.623, 0.646)) since $X_{i1}$ ($\alpha_{12}=0.75$) has a smaller effect on the type 2 event intensity function than $X_{i2}$ ($\alpha_{22}=1.5$). Moreover, regarding to evaluating the TRUE model, the proposed within‐event C‐index shows a lower discriminative ability on the type 1 event (Median = 0.791, IQR = (0.780, 0.801)) than on the type 2 event (Median = 0.827, IQR = (0.816, 0.836)). This is consistent with that the variance of the random effects of type 1 event is larger than that of the type 2 event.

## 6 APPLICATION TO THE NDS DATA

We fit the Bayesian multitype Poisson process model discussed in Section 3 to the Commercial Truck Driver NDS data. The models with different B‐spline settings are compared based on the assessment criteria developed in Section 4. The best fit model is first selected and further analysis under this model is then carried out in this section.

### 6.1 Model comparison

Each of the driving shifts is considered as a multitype event Poisson process with type 1 to 4 events representing ULD, CRC, SB, and LB, respectively. Dummy variables are created for the categorical variables, including the restarting group, the driver type, the starting time group, and the sleeping group. Following the findings from previous studies on the same subject,^9^, ^10^ we assume that each off‐duty sleeping time group has different patterns of intensity functions of the SCE risk as well as the on‐duty breaks incidence. Driving at 1:00 AM to 5:00 AM is considered as a time‐dependent covariate in the model. The rest of the driving shift level factors are assumed to have fixed effects on the intensity functions for different event types.

**FIGURE 6:**
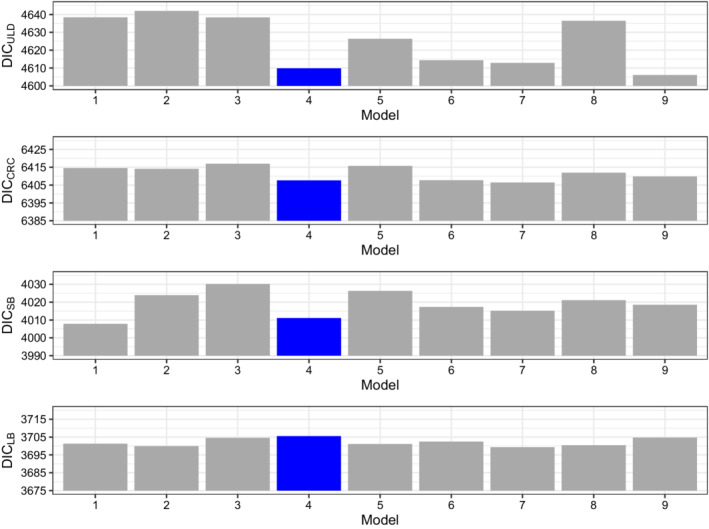
Comparing the type‐specific deviance information criterions among all of the candidate models

The candidate models are constructed by setting different parameters for the roughness penalty of the B‐splines, including the degree of basis splines (ν), the number of knots (k) and the order of the difference penalty (s). Quadratic (ν=2) and cubic (ν=3) spline functions with a first (s=1) or second (s=2) order penalty are considered in the candidate models. In all of the posterior computations, we take 5000 burn‐in samples, and 3000 MCMC samples with thinning of 10 iterations under each candidate model. Table 2 presents the values of the overall DIC and the LPML of the candidate models. We see from Table 2 that (i) the models with a smaller number of knots (K=5) have larger overall DICs and smaller LPMLs than the models with 10 or 15 knots; (ii) for the models with a larger number of knots (K=10 and K=15), with the same K, the models with cubic splines (ν=3) and the first order penalty (s=1) achieve larger overall DICs, and the models with more smoothed intensities (ν=3, s=2) have smaller LPMLs. This is consistent with the empirical results that both LPML and overall DIC have a good performance when the number of knots is under specified while LPML prefers a simpler model when the number of knots is over specified for the P‐splines. The slightly inconsistent model comparison results between DIC and LPML are also reported in the literature.^33^ In this data analysis, Model 4 (K=10,ν=3,s=1) has the second lowest overall DIC and the second highest LPML, indicating a good fit based on both overall DIC and LPML among all the candidate models. Thus, Model 4 is selected for further analysis. Moreover, the comparisons of the four type‐specific DICs are displayed in Figure 6. The differences of the type‐specific DICs between the candidate models are larger for the ULD and the SB events than the other two types of events. Specifically, the plots in Figure 6 indicate that (i) the differences of type‐specific DIC between the models are larger for ULD and SB compared with CRC and LB; (ii) Models 4 has relatively lower type‐specific DIC for ULD, CRC, and SB events simultaneously.

**TABLE 2:** Comparing deviance information criterion (DIC) and logarithm of the pseudo‐marginal likelihood (LPML) among all of the candidate models

Model	K	ν	s	DIC	LPML
1	5	3	1	18 762.1	−10 177.1
2	5	3	2	18 780.0	−10 222.5
3	5	2	1	18 790.1	−10 274.0
4	10	3	1	**18 734.1**	**−10 162.1**
5	10	3	2	18 769.5	**−10 129.2**
6	10	2	1	18 741.8	−10 254.7
7	15	3	1	**18 733.8**	−10 285.5
8	15	3	2	18 770.0	−10 185.3
9	15	2	1	18 739.1	−10 217.8

### 6.2 Posterior estimates under the selected model

To investigate the association of the incidence rates between SCEs and on‐duty breaks, Table 3 presents the posterior estimates of the parameters in the dependence structure of the joint model. A significant positive correlation is observed between ULDs and CRCs ($\rho_{12}$ = 0.892, 95% CI = [0.840, 0.933]), which is consistent with the results in Liu and Guo.^9^ It is interesting to see a significant positive correlation between ULD events and SBs ($\rho_{13}$ = 0.208, 95% CI = [0.019, 0.386]), which suggests that the driver's decision to take a SB is related to the fatigue level experienced in driving. When experiencing more ULD events due to fatigue, the driver may have a higher chance of taking a SB to mitigate fatigue. In addition, negative correlation between CRCs and LBs is obtained ($\rho_{24}$ = ‐0.472, 95%CI = [−0.803, −0.128]). This indicates that drivers may benefit from taking a long on‐duty break to improve driving safety.

**TABLE 3:** Posterior estimates of the covariance matrix of the random effects under Model 4

Mean (SD) 95% CI	ULD	CRC	SB	LB
ULD	2.124 (0.089)	0.892 (0.024)	0.208 (0.093)	−0.219 (0.190)
	[1.949, 2.295]	[0.840, 0.933]	[0.019, 0.386]	[−0.602, 0.139]
CRC		1.358 (0.059)	0.021 (0.103)	−0.472 (0.170)
		[1.245, 1.476]	[−0.180, 0.219]	[−0.803, ‐0.128]
SB			0.781 (0.082)	0.557 (0.231)
			[0.618, 0.936]	[0.033, 0.913]
LB				0.398 (0.090)
				[0.218, 0.578]

*Notes*: The diagonal elements report the estimates of the standard deviations in the dependence structure, and the upper‐diagonal elements present the estimates of the correlation between two types of events.

Abbreviations: CI, Credible interval; SD, Posterior standard deviation.

Table 4 presents the posterior estimates of the fixed effects of both time‐independent and time‐dependent covariates. Among the driving shifts in the “not restarting group," the drivers were more likely to take LB compared to the “restarting driving" group. For the intensity of LB, the posterior estimate of the coefficient for “not restarting driving" compared with “restarting driving" is 0.675 (95% CI = [0.140, 1.283]). This result indicates that drivers may take more long on‐duty breaks to compensate for the fatigue accumulated over a week‐long continuous working period. During the early morning period (1:00 AM‐5:00 AM), we see lower risks of ULD (−1.187, 95% CI = [−1.884, −0.522]) and CRC (−0.473, 95% CI = [−0.855, −0.110]) events compared to the other time of day. The light traffic condition could be the primary reason for the lower SCE risks during the early morning. Driving between 1:00 AM and 5:00 AM was positively associated with on‐duty LB (0.353, 95% CI = [0.020, 0.672]), which indicates that drivers may choose to have additional rest when driving in the late night. The starting time of a driving shift significantly impacted the short and LB intensities. Specifically, the driving shifts starting in the morning had a higher chance of taking both short and LB compared to those starting in the daytime (SB: 0.350, 95% CI = [0.107, 0.599]; LB: 0.410, 95% CI = [0.181, 0.636]) and at night (SB: 0.461, 95% CI = [0.148, 0.766]; LB: 0.702, 95% CI = [0.367, 1.046]). The number of nondriving tasks was associated with lower risk of ULD events (−0.203, 95% CI = [−0.376, −0.024]) as well as both lower SB (−0.233, 95% CI = [−0.361, −0.112]) and LB (−0.357, 95% CI = [−0.484, −0.226]) intensities. Thus, the nondriving tasks may be considered as a break from driving, which positively affects driving performance. In addition, the drivers with longer years of commercial license reported a lower risk of ULD (−0.228, 95% CI = [−0.402, −0.054]) and a lower chance of taking a SB (−0.127, 95% CI = [−0.245, −0.010]).

**TABLE 4:** Posterior estimates of the coefficients for the fixed effects in Model 4

Type	Coefficient	Mean	SD	95% CI
ULD	**Commercial licence year**	**−0.228**	**0.089**	**[−0.402, ‐0.054]**
	Driver type: long‐haul	−0.074	0.249	[−0.640, 0.361]
	**Number of nondriving work**	**−0.203**	**0.090**	**[** −0.376, −0.024 **]**
	Starting a shift in the morning vs daytime	−0.033	0.181	[−0.377, 0.338]
	Starting a shift at night vs daytime	−0.214	0.225	[−0.666, 0.221]
	Starting a shift in the morning vs night	0.180	0.243	[−0.330, 0.607]
	Restarting driving vs no need to restart	0.367	0.351	[−0.325, 1.046]
	Not restarting driving vs no need to restart	0.121	0.186	[−0.245, 0.503]
	Not restarting driving vs restarting driving	−0.247	0.363	[−0.917, 0.481]
	**Driving between 1:00 AM and 5:00 AM**	**−1.187**	**0.341**	**[−1.884, ‐0.522]**
CRC	Commercial licence year	−0.099	0.056	[−0.205, 0.017]
	Driver type: long‐haul	−0.044	0.137	[−0.317, 0.222]
	Number of non‐driving work	−0.062	0.055	[−0.169, 0.045]
	Starting a shift in the morning vs daytime	−0.098	0.120	[−0.336, 0.134]
	**Starting a shift at night vs daytime**	**−0.271**	**0.146**	**[−0.554, ‐0.002]**
	Starting a shift in the morning vs night	0.174	0.156	[−0.144, 0.457]
	Restarting driving vs no need to restart	0.231	0.242	[−0.244, 0.690]
	Not restarting driving vs no need to restart	−0.041	0.121	[−0.279, 0.198]
	Not restarting driving vs restarting driving	−0.272	0.250	[−0.751, 0.211]
	**Driving between 1:00 AM and 5:00 AM**	**−0.473**	**0.192**	**[‐0.855, ‐0.110]**
SB	**Commercial licence year**	**−0.127**	**0.059**	**[−0.245, ‐0.010]**
	Driver type: long‐haul	−0.230	0.185	[−0.596, 0.123]
	**Number of nondriving work**	**−0.233**	**0.063**	**[−0.361, ‐0.112]**
	**Starting a shift in the morning vs daytime**	**0.350**	**0.123**	**[0.107, 0.599]**
	Starting a shift at night vs daytime	−0.111	0.166	[−0.434, 0.209]
	**Starting a shift in the morning vs night**	**0.461**	**0.157**	**[0.148, 0.766]**
	Restarting driving vs no need to restart	−0.371	0.288	[−0.955, 0.185]
	Not restarting driving vs no need to restart	−0.187	0.130	[−0.442, 0.070]
	Not restarting driving vs restarting driving	0.187	0.298	[−0.383, 0.789]
	Driving between 1:00 AM and 5:00 AM	−0.242	0.202	[−0.655, 0.154]
LB	Commercial licence year	0.030	0.047	[−0.067, 0.120]
	**Driver type: long‐haul**	**1.383**	**0.348**	**[0.716, 2.068]**
	**Number of non‐driving work**	**−0.357**	**0.065**	**[−0.484, ‐0.226]**
	**Starting a shift in the morning vs daytime**	**0.410**	**0.118**	**[0.181, 0.636]**
	Starting a shift at night vs daytime	−0.291	0.176	[−0.645, 0.045]
	**Starting a shift in the morning vs night**	**0.702**	**0.172**	**[0.367, 1.046]**
	Restarting driving vs no need to restart	−0.465	0.292	[−1.081, 0.060]
	Not restarting driving vs no need to restart	0.210	0.111	[−0.006, 0.424]
	**Not restarting driving vs restarting driving**	**0.675**	**0.296**	**[0.140, 1.283]**
	**Driving between 1:00 AM and 5:00 AM**	**0.353**	**0.168**	**[0.020, 0.672]**

Abbreviations: CI, Credible interval; SD, Posterior standard deviation.

**FIGURE 7:**
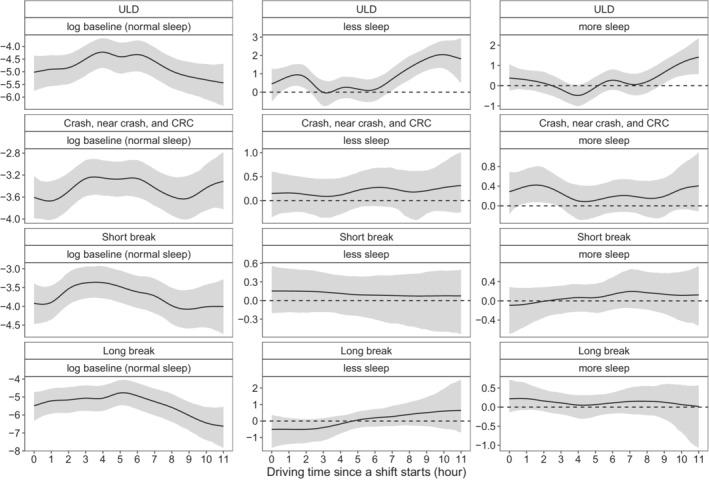
Time‐varying effect of the off‐duty sleeping groups on the intensity function of unintentional lane deviations, crash‐relevant conflict, short breaks, and long breaks events

The estimated smoothing curves of the baseline intensities and the time‐varying coefficients are presented in Figure 7. Similar to the results in Liu and Guo,^9^ we observe an increase of the intensity rate of ULD events in each of the insufficient sleeping group and the abundant sleeping group. However, there are no obvious time‐varying effects of the sleeping groups on the intensity functions of the on‐duty break events.

**FIGURE 8:**
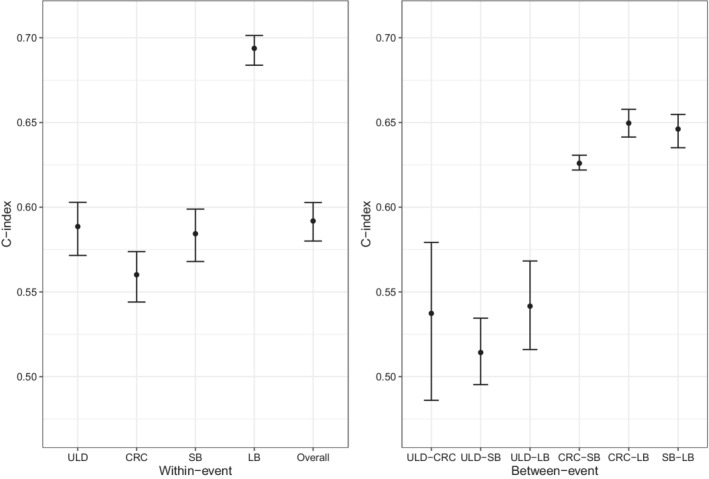
Posterior estimates of the within‐event C‐indices, the between‐event C‐indices, and the overall C‐index for Model 4

As shown in Figure 8, we calculate the proposed C‐indices for the muti‐type recurrent event model to evaluate the selected model's adequacy on the four types of recurrent events. In general, Model 4 fits the data moderately well with an overall C‐index of 0.592 (95% CI = [0.580, 0.603]). Model 4 has a relatively high discrimination ability on LB with the within‐event C‐index 0.694 (95% CI = [0.684, 0.701]). The within‐event C‐indices for ULD, CRC, and SBs are 0.589 (95% CI = [0.572, 0.603]), 0.560 (95% CI = [0.544, 0.574]), and 0.584 (95% CI = [0.568, 0.599]), respectively, which are lower than that for the LB events. These results are consistent with the the posterior estimates of the parameters in Model 4. The model has more significant fixed effects and a smaller variance of random effect for LB events, which results in a relatively higher discrimination ability on the LB events.

**FIGURE 9:**
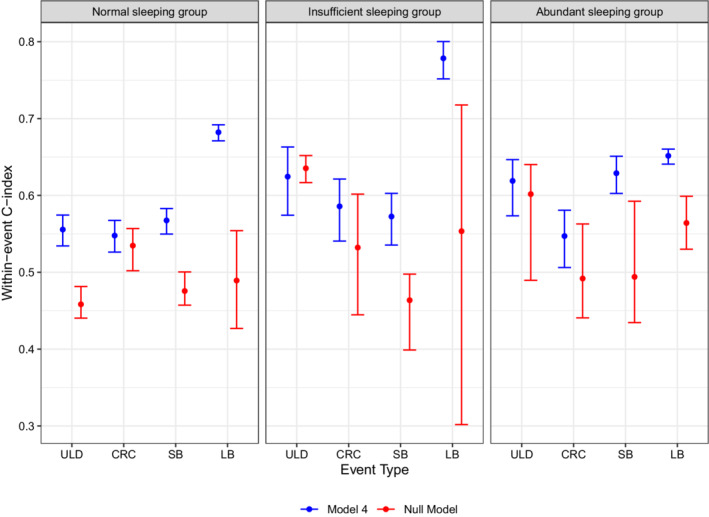
Comparing the within‐event C‐indices between the Model 4 and a null model within each of the sleeping groups

As displayed in Figure 9, we further compare the C‐indices between Model 4 and a null model having the off‐duty sleeping groups as the only predictor with time‐varying effects. Within each of the three sleeping groups, Model 4 gains an increase of the within‐event C‐index for both short and LB by including the fix effects of both time‐invariant and time‐dependent covariates, since many of these predictors are significant to the intensity of SB and LB as shown in Table 4. The within‐event C‐indices for the two SCE events of the two models are similar.

## 7 DISCUSSION

In this paper, we have developed several model assessment criteria for evaluating and comparing the adequacy of the Bayesian multitype recurrent event models. Our proposed approaches are attractive in assessing the goodness‐of‐fit of the models in different aspects: (i) the easily computed type‐specific DICs assess the model fit for each type of the recurrent events, and (ii) the overall DIC and the LPML capture the model fit for all types of the recurrent events simultaneously. Unlike the DIC, the type‐specific decomposition of the LPML is not straightforward but possible by following the LPML decomposition under the joint models for longitudinal and survival data.^15^, ^34^ However, such decomposition requires substantial theoretical derivations and new developments of computational algorithms.

The proposed C‐indices are able to evaluate the discrimination abilities of the Bayesian multi‐type recurrent event models in the different angles—that is, within‐event C‐index, between‐event C‐index, and overall C‐index. Compared with the existing C‐index for recurrent event models,^19^ the proposed method is still easy to implement under the complex multi‐type recurrent model with a large number of random effects, time‐varying coefficients and time‐dependent predictors since the comparable pairs are constructed based on the event rates from two event processes. We note that the independence between the termination of the event process and the occurrence of events is required, which is proper in the NDS application since the end of a driving task is usually prescheduled rather than based on SCEs or driving behaviors occurring in a driving shift.

This study is among the first to jointly model drivers' rest break behaviors and safety outcomes with consideration of several key risk factors, such as driving between 1:00 AM and 5:00 AM, week‐long cumulative driving hours,and driving shift starting time. The results indicate that driving between 1:00 AM and 5:00 AM is associated with significantly lower ULD and SCE intensity. The 1:00 AM to 5:00 AM period is a critical rest time due to the circadian rhythm and it is generally suggested that sleeping during this time is important for reducing fatigue. However, many truck drivers prefer driving at night to avoid traffic congestion as well as being constrained by a delivery schedule. A truck driver can adjust their circadian rhythm to mitigate the fatigue associated with night time driving. The low traffic volume during the 1:00 AM to 5:00 AM period represents less vehicle interaction and thus a relatively safe driving environment, which could lead to the relatively low ULD and CRC during this time. A comprehensive evaluation of the benefit and risk is needed to provide a safety strategy for driving at this critical period.

The results also imply drivers tend to take various countermeasures to mitigate fatigue. When they could not take a 34‐hour break after 60 hours over 7 consecutive days or 70 hours over 8 consecutive days as recommended by the HOS rule, the drivers were more likely to to take LB while driving. Similarly, the intensity of LB was higher during the 1:00 AM to 5:00 AM period. When taking more work‐related breaks, drivers tended to have fewer rest breaks, both long and short. These results imply the self‐adaptation of drivers in managing their schedule to improve driving safety. A systematic fatigue management approach using a combination of various methods will mitigate fatigue while driving and benefit driving safety for commercial truck drivers.

## Supporting information




**Table S1**. Summary of the DIC and LPML differences between TRUE and each of the misspecified models in Simulation Study 1. Table S2. Summary of the C‐indices of the fitted models in Simulation Study 2. Figure S1. True curves of the baseline intensity functions and time‐varying coefficients. Figure S2. Coverage probability of the baseline intensity functions and time‐varying coefficients under different choices of $a_{\tau }$

## Data Availability

The data that support the findings of this study are available from the Virginia Tech Transportation Institute. Restrictions apply to the availability of these data, which were used under license for this study. Data are available from the corresponding author with the permission of the Virginia Tech Transportation Institute.

## APPENDIX A Derivation of the normalized weight function

### A.1

As introduced in Section 4.2, the normalized weight function $w_{i}$ is chosen by a multivariate normal density constructed by the Laplace approximation of the joint density (14). We write that $\Lambda_{ij}(c_{i})={\tilde{\Lambda }}_{ij}(c_{i})\exp(b_{ij})$ and $\pi_{ij}(t)=\exp\{X_{i}^{\prime }\alpha_{j}+W_{i}{\left(t\right)}^{\prime }\gamma_{j}+\beta_{0j}(t)+Z_{i}^{\prime }\beta_{j}(t)\}$. Then, the joint density (14) can be written by

$$\begin{array}{llll} f(t_{i},b_{i}|\Gamma ,D_{i}) & =\overset{J}{\underset{j=1}{\prod }}\left\{\overset{n_{ij}}{\underset{k=1}{\prod }}\{\pi_{ij}(t_{ijk})\exp(b_{ij})\}\exp(-{\tilde{\Lambda }}_{ij}(c_{i})\exp(b_{ij}))\right\}\; & & \\ & \;\times \frac{1}{{\left(2\pi \right)}^{\frac{J}{2}}|\sum {|}^{\frac{1}{2}}}\exp\{-\frac{1}{2}b_{i}^{T}\sum^{-1}b_{i}\}\; & & \\ & =\overset{J}{\underset{j=1}{\prod }}\left\{\overset{n_{ij}}{\underset{k=1}{\prod }}\pi_{ij}(t_{ijk})\right\}\; & & \\ & \;\times \frac{1}{{\left(2\pi \right)}^{\frac{J}{2}}|\sum {|}^{\frac{1}{2}}}\exp\left\{\overset{J}{\underset{j=1}{\sum }}n_{ij}b_{ij}-\overset{J}{\underset{j=1}{\sum }}{\tilde{\Lambda }}_{ij}(c_{i})\exp(b_{ij})-\frac{1}{2}b_{i}^{T}\sum^{-1}b_{i}\right\}\; & & \\ & =\frac{C_{i}}{{\left(2\pi \right)}^{\frac{J}{2}}|\sum {|}^{\frac{1}{2}}}\exp(h(b_{i})),\; & & \end{array}$$

where

$$C_{i}=\overset{J}{\underset{j=1}{\prod }}\{\overset{n_{ij}}{\underset{k=1}{\prod }}\pi_{ij}(t_{ijk})\}\;\text{and}$$

$$h(b_{i})=\overset{J}{\underset{j=1}{\sum }}n_{ij}b_{ij}-\overset{J}{\underset{j=1}{\sum }}{\tilde{\Lambda }}_{ij}(c_{i})\exp(b_{ij})-\frac{1}{2}b_{i}^{T}\sum^{-1}b_{i}.$$

Let $b_{i0}$ denote the mode of $h(b_{i})$ by solving $\frac{\partial h(b_{i})}{\partial b_{i}}=0$. In practice, the equation can be solved numerically. By the Laplace approximation, we can get

$$\begin{array}{llll} f(t_{i},b_{i}|\Gamma ,D_{i}) & \approx \frac{C_{i}}{{\left(2\pi \right)}^{\frac{J}{2}}|\sum {|}^{\frac{1}{2}}}\exp\left(h(b_{i0})+\frac{1}{2}{\left(b_{i}-b_{i0}\right)}^{T}\frac{\partial^{2}h(b_{i0})}{\partial b_{i}\partial b_{i}^{T}}(b_{i}-b_{i0})\right)\; & & \\ & =\frac{C_{i}\exp(h(b_{i0}))}{|\sum {|}^{\frac{1}{2}}}\times \frac{1}{{\left(2\pi \right)}^{\frac{J}{2}}}\exp\left(\frac{1}{2}{\left(b_{i}-b_{i0}\right)}^{T}\frac{\partial^{2}h(b_{i0})}{\partial b_{i}\partial b_{i}^{T}}(b_{i}-b_{i0})\right),\; & & \end{array}$$

and the elements in the Hessian matrix $\frac{\partial^{2}h(b_{i})}{\partial b_{i}\partial b_{i}^{T}}$ are given by

$$\frac{\partial^{2}h}{\partial b_{ij}^{2}}=-{\tilde{\Lambda }}_{ij}(c_{i})\exp(b_{ij})-\sigma_{jj}\;\text{and}\;\frac{\partial^{2}h}{\partial b_{ij}b_{ik}}=-\sigma_{kj},$$

where $\sigma_{kj}$ is the entry of row k and column j of $\sum^{-1}$. Therefore, we set up the $w_{i}(b_{i})$ as

$$w_{i}(b_{i})=\phi (b_{i};\tilde{\mu },\tilde{\sum }).$$

Let ϕ denote the normal density function with mean $\tilde{\mu }$ and covariance matrix $\tilde{\sum }$, where $\tilde{\mu }=b_{i0}$ and $\tilde{\sum }={\left(-\frac{\partial^{2}h(b_{i0})}{\partial b_{i}\partial b_{i}^{T}}\right)}^{-1}$.
