# Supplementary material for: Bayesian criterion‐based assessments of recurrent event models with applications to commercial truck driver behavior studies
Source: Stat Med. 2022 Jul 24;41(23):4607–28. doi: 10.1002/sim.9528 (PMC9796651; doi:10.1002/sim.9528)
Supplement: Supplementary file 1 — Table S1. Summary of the DIC and LPML differences between TRUE and each of the misspecified models in Simulation Study 1. Table S2. Summary of the C‐indices of the fitted models in Simulation Study 2. Figure S1. True curves of the baseline intensity functions and time‐varying coefficients. Figure S2. Coverage probability of the baseline intensity functions and time‐varying coefficients under different choices of aτ [file SIM-41-4607-s001.pdf]

Bayesian Criterion-based Assessments of Recurrent Event Models with Applications to Commercial Truck Driver Behavior Studies Supplementary Materials

Yiming Zhang, Ming-Hui Chen, Feng Guo

S.1 Additional Tables

Table S.1: Summary of the DIC and LPML differences between TRUE and each of the misspecified models in Simulation Study 1.

Model	Type-1 Event DIC Difference			Type-2 Event DIC Difference		
	Median	IQR	% of <0	Median	IQR	% of <0
MoreK10	-0.039	(-4.303, 3.385)	52%	0.725	(-2.584, 3.636)	46%
LessK3	-6.652	(-12.573, -1.288)	82%	-5.767	(-10.352, -1.450)	84%
MissZ2	-16.374	(-24.700, -9.941)	96%	-8.376	(-14.620, -4.160)	91%
MissX	-2.737	(-6.406, 0.571)	71%	-1.647	(-4.701, 0.706)	66%
MissXZ2	-18.643	(-27.612, -13.204)	98%	-11.195	(-17.375, -4.531)	89%
Model	Overall DIC Difference			LPML Difference		
	Median	IQR	% of <0	Median	IQR	% of >0
MoreK10	1.102	(-5.787, 5.477)	44%	0.776	(-0.576, 2.336)	67%
LessK3	-13.447	(-21.586, -4.348)	91%	6.264	(3.186, 10.150)	94%
MissZ2	-26.329	(-36.481, -16.525)	98%	13.749	(9.768, 17.911)	99%
MissX	-4.472	(-8.780, -0.952)	79%	8.37	(5.988, 12.660)	99%
MissXZ2	-30.639	(-40.449, -21.65)	99%	23.265	(17.875, 27.972)	100%

Table S.2: Summary of the C-indices of the fitted models in Simulation Study 2.

Model	Type-1 Within-event C-index		Type-2 Within-event C-index	
	Median	IQR	Median	IQR
TRUE	0.791	(0.780, 0.801)	0.827	(0.816, 0.836)
MissX1	0.630	(0.616, 0.641)	0.787	(0.777, 0.797)
MissX2	0.756	(0.744, 0.766)	0.635	(0.623, 0.646)
MissX1X2	0.537	(0.517, 0.546)	0.515	(0.504, 0.532)
Model	Between-event C-index		Overall C-index	
	Median	IQR	Median	IQR
TRUE	0.804	(0.795, 0.811)	0.806	(0.797, 0.814)
MissX1	0.678	(0.667, 0.696)	0.694	(0.682, 0.704)
MissX2	0.703	(0.692, 0.714)	0.699	(0.688, 0.708)
MissX1X2	0.542	(0.526, 0.551)	0.532	(0.524, 0.541)

S.2 Additional Figures

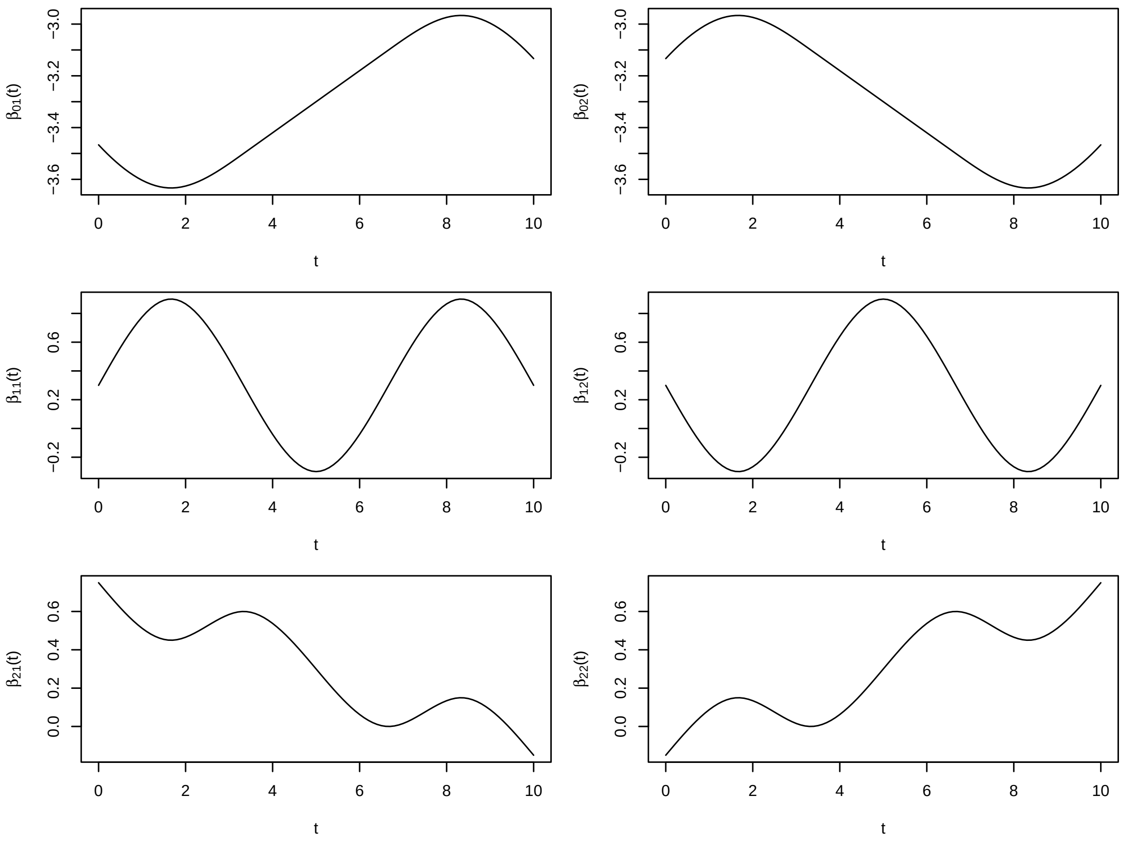

Figure S.1: True curves of the baseline intensity functions and time-varying coefficients.

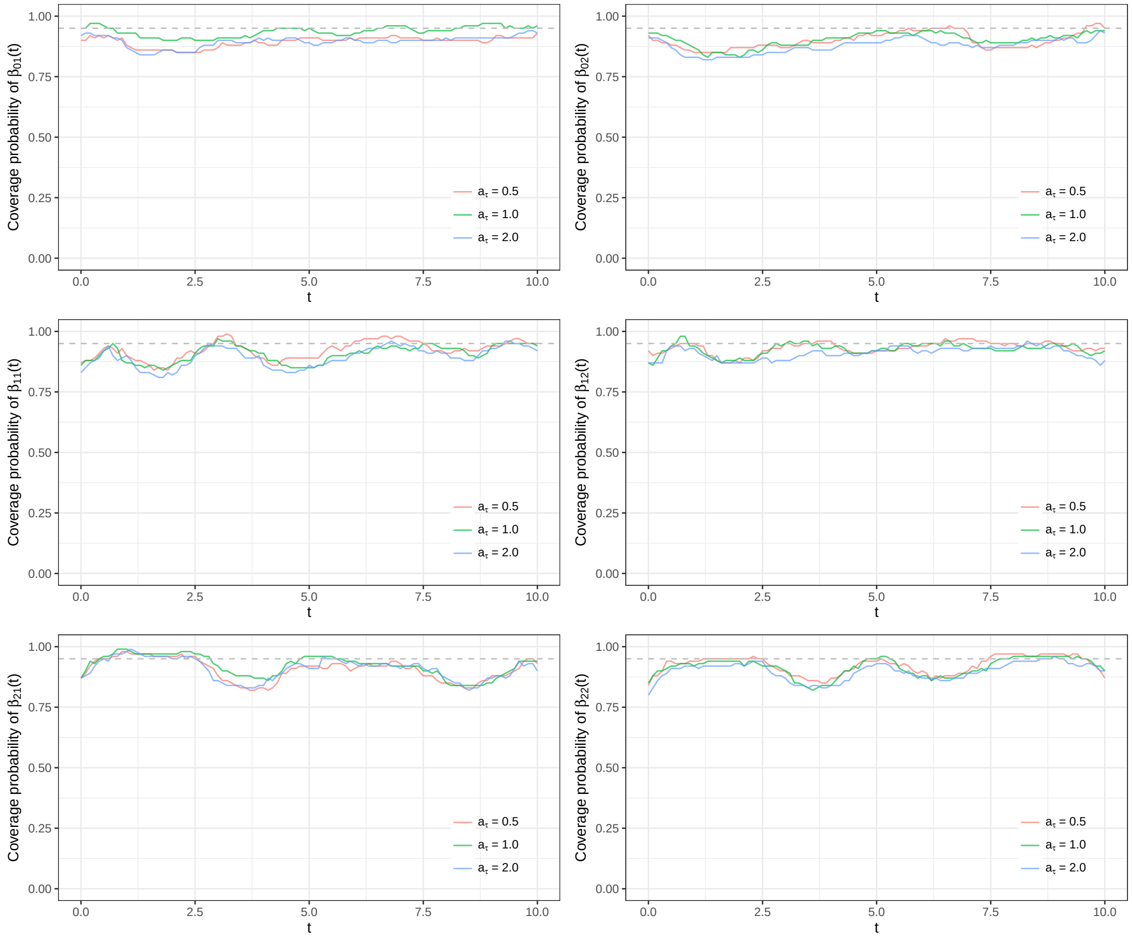

Figure S.2: Coverage probability of the baseline intensity functions and time-varying coefficients under different choices of a_τ.
